# Soluble Epoxide Hydrolase Is Associated with Postprandial Anxiety Decrease in Healthy Adult Women

**DOI:** 10.3390/ijms231911798

**Published:** 2022-10-05

**Authors:** Nhien Nguyen, Christophe Morisseau, Dongyang Li, Jun Yang, Eileen Lam, D. Blake Woodside, Bruce D. Hammock, Pei-an Betty Shih

**Affiliations:** 1 Department of Psychiatry, University of California San Diego, San Diego, CA 92037, USA; 2 Department of Entomology and Nematology and Comprehensive Cancer Center, University of California, Davis, CA 95616, USA; 3 Centre for Mental Health, University Health Network, Toronto, ON M5G 2C4, Canada

**Keywords:** soluble epoxide hydrolase, healthy adults, anxiety, metabolism

## Abstract

The metabolism of bioactive oxylipins by soluble epoxide hydrolase (sEH) plays an important role in inflammation, and sEH may be a risk modifier in various human diseases and disorders. The relationships that sEH has with the risk factors of these diseases remain elusive. Herein, sEH protein expression and activity in white blood cells were characterized before and after a high-fat meal in healthy women (HW) and women with anorexia nervosa (AN). sEH expression and sEH activity were significantly correlated and increased in both groups two hours after consumption of the study meal. Fasting sEH expression and activity were positively associated with body mass index (BMI) in both groups, while an inverse association with age was found in AN only (*p* value < 0.05). sEH was not associated with anxiety or depression in either group at the fasting timepoint. While the anxiety score decreased after eating in both groups, a higher fasting sEH was associated with a lower postprandial anxiety decrease in HW (*p* value < 0.05). sEH characterization using direct measurements verified the relationship between the protein expression and in vivo activity of this important oxylipin modulator, while a well-controlled food challenge study design using HW and a clinical control group of women with disordered eating elucidated sEH’s role in the health of adult women.

## 1. Introduction

Cytochrome P450 (CYP) oxidizes polyunsaturated fatty acids (PUFA) to yield oxylipin products, termed epoxy fatty acids (epoxides, or EpFAs), which are inflammation-resolving bioactive lipid mediators [1,2,3]. Soluble epoxide hydrolase (sEH) converts these useful oxylipins into more polar, easily conjugated and excreted dihydroxy (diol) metabolites that lack the anti-inflammatory activity of the epoxides and, in some cases, can be pro-inflammatory [1,2,3,4,5,6]. The sEH is found in numerous organs, mostly in the liver and the kidney, in both the cytosol and peroxisomes [7,8]. It is encoded by the *Epoxide Hydrolase 2* (*EPHX2*) gene, mapped on 8p21.2–p21.1 [7,9,10,11,12,13,14,15]. Although sEH is a key modulator of CYP bioactive lipids, hence, playing a key role in inflammation processes, little is known about the relationship between sEH protein expression and in vivo activity, or how they affect phenotypes in human health.

The interplay between sEH and human diet is evident, as n-3 and n-6 PUFA, which are mainly obtained from dietary sources, undergo the CYP pathway to form the direct substrates of sEH, epoxides [1,2,3,4,5,6]. Several studies have demonstrated a significant relationship between food intake and sEH, including a study using an sEH in vivo activity marker (diol/epoxide ratios) that showed sEH was significantly reduced after rats ate diets with various potassium levels [16]. Another study found *EPHX2* mRNA expression to be increased in overweight/obese individuals after eating a meal high in fat [17]. Several other studies using animal models have observed that a high-fat diet is associated with sEH upregulation, while a low-fat diet may lower sEH levels [18,19,20]. The effects of a high-fat diet on sEH in humans, however, remain unclear.

High-fat diets can trigger inflammatory processes that underlie the pathogenesis of several human disorders/diseases such as cardiometabolic disorders [21,22], thus, making diet one of the most important modifiable risk factors clinically. The involvement of sEH in lipid metabolism and inflammation thus implicates sEH as a potential risk modifier in various diseases. Previous studies have suggested that sEH inhibition may confer protective effects in Alzheimer’s disease [23], Parkinson’s disease [24], cardiovascular diseases [25,26], preeclampsia [27], and inflammatory bowel disease [26], rendering sEH inhibition a potential treatment target.

Similar to unhealthy diet, age and body mass index (BMI) are key risk factors for many disorders involving inflammation, including cardiometabolic [28,29,30,31,32], cancer [33,34], preeclampsia [35], and psychiatric conditions [30,36,37,38]. Aging is a biological process that increases the risk for almost all health-related adverse phenotypes [28,29,30,31,32,33,35,37], while BMI is a measure of obesity, which has been linked to an increased risk for cardiometabolic disorders and death [28,31,32,39,40]. While the relationships of sEH with obesity [9,41,42,43,44,45,46,47,48,49] and age [50,51,52] have been indirectly assessed in several animal studies, how age and BMI affect sEH in adult women has not been directly examined.

Anxiety and depression are prevalent psychological disorders that have also been linked to inflammation and lipid alterations [53,54,55,56,57]. Previous studies using patients with depression and animal models of depression have reported elevated sEH under chronic stress and depressive conditions [58,59,60]. An sEH knock-out model and sEH downregulation have both shown anti-depressive and anxiety-modulating effects in animal models [59,60,61,62,63,64], implying that sEH may contribute to the etiology of depression and anxiety.

To research sEH, most published studies employed oxylipins, specifically CYP diol/epoxide ratios, as proxy markers of sEH in vivo activity. While oxylipins are important bioactive lipids themselves, the limitations of using proxy markers to research sEH include confounding effects introduced by variability in the bioavailability of the precursor fatty acid, as well as factors affecting metabolic pathways such as beta-oxidation, phospholipid remodeling, and enzymatic competition [65,66,67]. Direct measurement remains the gold standard for biomarker studies.

While multiple studies have implicated she as an associated “biomarker” for a number of “diseases/disorders”, the knowledge of how sEH associates with the risk factors (high-fat diet, age, and BMI) of these diseases/disorders is necessary to accurately assess the contribution of sEH and related oxylipins to these disorders. We leveraged three elements, direct sEH protein expression and activity measurements, a food challenge protocol, and the use of a clinical control arm to examine the relationships that sEH has with these risk factors in two groups of adult women.

## 2. Results

### 2.1. Characteristics of Study Participants

Healthy women (HW) and women with anorexia nervosa (AN, the clinical control group) were used as study subjects. Of the 96 HW (18–53 years old; mean age: 29 ± 8) with no known major medical or psychiatric disorders, 48 (50%) self-identified as White, 39 (41%) as Asian, two (2%) as Black or African American, and seven (7%) as “more than one race” (Table 1). The AN group consisted of 70 women (18–68 years old; mean age: 32 ± 12) with a confirmed diagnosis of AN, a common form of eating disorder characterized by the avoidance of foods—especially those of high-fat content—in order to lose weight or maintain an extremely low weight. Sixty-two women (89%) in this group identified themselves as White, seven (10%) as Asian, and one (1%) as “more than one race” (Table 1). The HW group contained a lower proportion of White women and a higher proportion of Asian women compared to the AN group (*p* < 0.0001) (Table 1).

No significant difference in age was observed between HW and women with AN. The two groups differed significantly in five anthropometric and clinical measurements. The healthy women group exhibited a significantly higher mean body mass index (22.98 ± 3.16 versus 18.66 ± 3.44, *p* value < 0.0001); lower depression level (4.05 ± 6.22 versus 22.900 ± 15.75, *p* value < 0.0001); lower anxiety at both fasting (4.74 ± 7.06 versus 22.27 ± 13.38, *p* value < 0.0001) and postprandial timepoints (3.64 ± 6.50 versus 15.82 ± 11.50, *p* value < 0.0001); and a smaller postprandial unit decrease in anxiety (−1.20 ± 2.83 versus −4.32 ± 7.23, *p* value = 0.0005) compared to the AN group (Table 1).

### 2.2. Relationship between sEH Expression and sEH Activity

**Figure 1:**
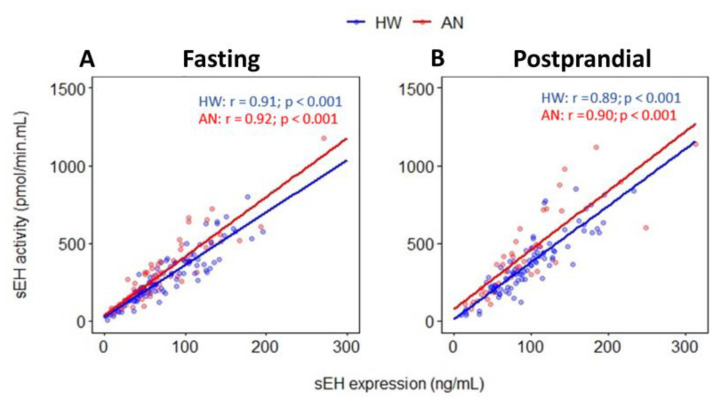
Pearson’s correlations between soluble epoxide hydrolase (sEH) expression and sEH activity in healthy women (blue) and women with AN (red) at (**A**) fasting and (**B**) postprandial timepoints. Colored dots and solid lines represent individual data points and slopes of the examined relationships, respectively. r and p indicate Pearson’s correlation coefficient and *p* value, respectively. HW: healthy women; AN: anorexia nervosa.

At the fasting timepoint, sEH protein expression exhibited a positive correlation with sEH activity in both HW and AN groups (*p* value < 0.001) (Figure 1A). Similarly, at the postprandial timepoint, sEH expression and activity remained positively correlated in both groups (*p* value < 0.001) (Figure 1B). After adjustment for age and BMI, HW displayed an increase of 3.36 pmol/min.mL in sEH activity per every one-unit increase in sEH expression (95% CI: 3.04 to 3.68 pmol/min.mL; *p* value < 0.001) at fasting. In the AN group, for each unit increase in sEH expression, sEH activity was upregulated by 3.70 pmol/min.mL (95% CI: 3.23 to 4.17 pmol/min.mL; *p* value < 0.001). At the postprandial timepoint, among HW, for each unit increase in sEH expression, sEH activity increased by 3.63 pmol/min.mL (95% CI: 3.23 to 4.04 pmol/min.mL; *p* value < 0.001). Similarly, in the AN group, for every one-unit increase in sEH expression, sEH activity increased by 3.62 pmol/min.mL (95% CI: 2.69 to 4.55 pmol/min.mL; *p* value < 0.001). Cell-count normalized sEH expression and activity demonstrated significantly high correlations with absolute sEH expression and activity in both groups (*p* value ≤ 1.166 × 10^−6^).

### 2.3. Effect of a High-Fat Meal on sEH Levels

**Figure 2:**
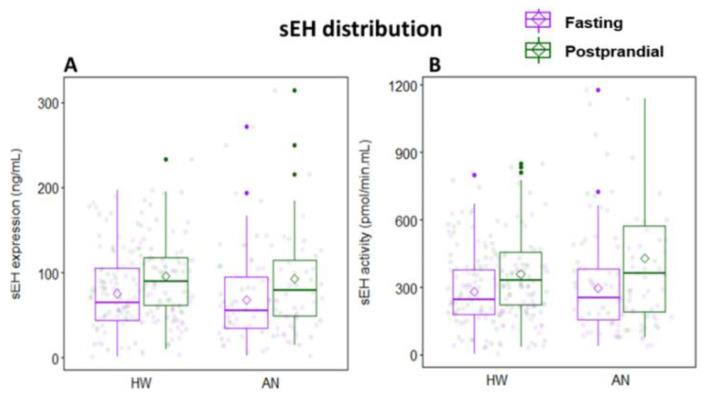
Distribution of (**A**) sEH expression and (**B**) sEH activity in healthy women and women with anorexia nervosa at fasting (purple) and postprandial (green) timepoints. Diamonds and colored dots represent means and individual data points, respectively. Boxplots indicate the 25th percentile, median, and 75th percentile. HW: healthy women; AN: anorexia nervosa.

**Figure 3:**
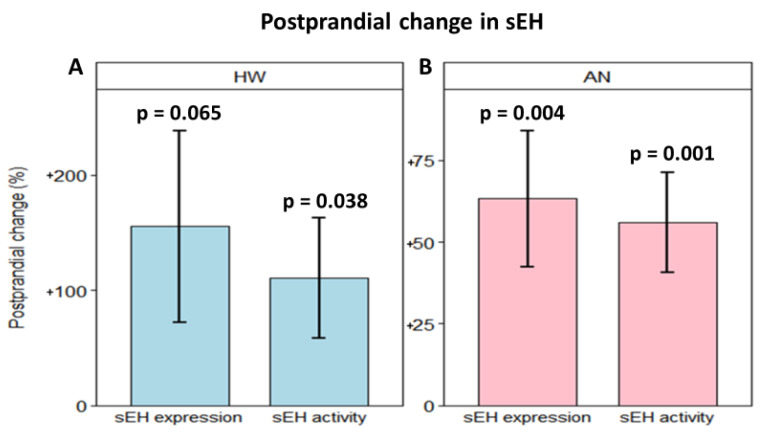
Bar plots of postprandial changes (%) in sEH expression and sEH activity in (**A**) healthy women and (**B**) women with anorexia nervosa at 2-h after consumption of the study meal. Bars and error bars represent the mean and standard error of the mean, respectively. p represents the *p* value of one-sample *t*-test for each study group, separately. HW: healthy women; AN: anorexia nervosa.

At the fasting timepoint, the HW group had a mean sEH expression of 75.70 ± 43.97 ng/mL (95% CI: 66.79 to 84.60 ng/mL, Figure 2A) and a mean sEH activity of 281.80 ± 163.62 pmol/min.mL (95% CI: 248.65 to 314.95 pmol/min.mL, Figure 2B). Two hours after consumption of the high-fat study sandwich, sEH expression increased by 19.14 ± 41.07 ng/mL (95% CI: 10.64 to 27.65 ng/mL), equivalent to a 155.44% within-group increase (SD: 798.16%, *p* value = 0.065) in HW (Figure 3A). sEH activity increased by 76.98 ± 157.17 pmol/min.mL (95% CI: 6.45 to 214.75 pmol/min.mL), equivalent to a 110.60% within-group increase (SD: 502.92%, *p* value = 0.038) in HW (Figure 3B).

**Figure 4:**
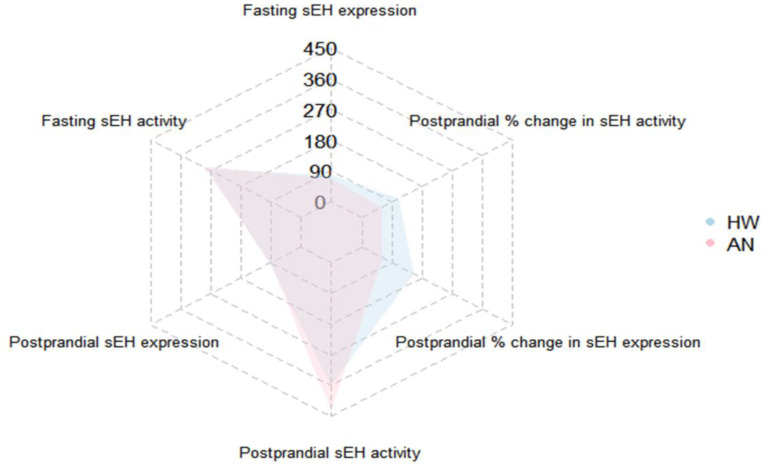
Polygonal graph summarizing mean sEH levels at fasting and postprandial timepoints and mean postprandial sEH changes (%) in healthy women (blue) and women with anorexia nervosa (pink). HW: healthy women; AN: anorexia nervosa.

In the AN group, fasting mean sEH expression was 67.96 ± 48.28 ng/mL (95% CI: 56.44 to 79.47 ng/mL, Figure 2A), and mean sEH activity was 295.15 ± 200.67 pmol/min.mL (95% CI: 247.31 to 343.00 pmol/min.mL, Figure 2B). Two hours after eating, sEH expression was elevated by 17.92 ± 46.00 ng/mL (95% CI: 2.80 to 33.04 ng/mL), equivalent to a 63.28% within-group increase (SD: 128.55%, *p* value = 0.004) in women with AN (Figure 3A). sEH activity increased by 101.71 ± 201.65 pmol/min.mL (95% CI: 25.10 to 87.02 pmol/min.mL), equivalent to a 56.06% within-group increase (SD: 94.12%, *p* value = 0.001) in AN (Figure 3B). A polygonal graph presenting a summary of sEH under various conditions is shown in Figure 4. An assessment of sEH expression and activity using cell-count normalized values yielded no significant differences in the data shown in Figure 2.

### 2.4. Associations of sEH with Age and Body Mass Index (BMI)

**Figure 5:**
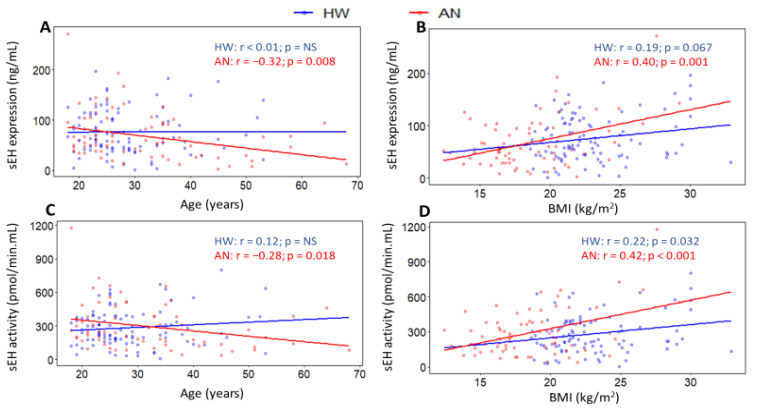
Scatterplots for Pearson’s correlations of (**A**,**B**) fasting sEH expression and (**C**,**D**) sEH activity with age and BMI in healthy women (blue) and women with anorexia nervosa (red). Colored dots and solid lines represent individual data points and slopes of the examined relationships, respectively. r and p indicate Pearson’s correlation coefficient and *p* value, respectively. HW: healthy women; AN: anorexia nervosa; BMI: body mass index; NS: non-significant, *p* value ≥ 0.10.

In HW, age was not significantly correlated with either sEH expression (*p* value = 0.980) or sEH activity (*p* value = 0.262) (Figure 5A,C). Age was also not significantly correlated with postprandial changes in either sEH activity or expression. By contrast, in women with AN, age was significantly and inversely correlated with both sEH expression (r = −0.32; *p* value = 0.008) and sEH activity (r = −0.28; *p* value = 0.018) (Figure 5A,C). After BMI adjustment, for every additional year in age, sEH expression decreased by 1.18 ng/mL (95% CI: −2.06 to −0.30 ng/mL; *p* value = 0.009), while sEH activity reduced by 4.30 pmol/min.mL (95% CI: −7.95 to −0.65 pmol/min.mL; *p* value = 0.022). Age was positively correlated with postprandial percent changes of both sEH expression (r = 0.46; *p* value = 0.004) and sEH activity (r = 0.32; *p* value = 0.05). When BMI was adjusted for, each additional year in age was associated with postprandial increases of 4.83% in sEH expression (95% CI: 1.54 to 8.12%; *p* value = 0.005) and 2.33% in sEH activity (95% CI: −0.22 to 4.87%; *p* value = 0.072).

In HW, BMI showed a positive correlation with sEH expression (r = 0.19; *p* value = 0.067) and sEH activity (r = 0.22; *p* value = 0.032) (Figure 5B,D). When controlling for age, for every unit increase in BMI, sEH expression and activity increased by 2.81 ng/mL (95% CI: −0.11 to 5.72 ng/mL; *p* value = 0.059) and 10.51 pmol/min.mL (95% CI: −0.27 to 21.29 pmol/min.mL; *p* value = 0.056), respectively. In the AN group, BMI was also positively correlated with both sEH expression (r = 0.40; *p* value = 0.001) and sEH activity (r = 0.42; *p* value = 0.0003) (Figure 5B,D). After age adjustment, for each unit increase in BMI, sEH expression and activity increased by 5.24 ng/mL (95% CI: 2.25 to 8.23 ng/mL; *p* value = 0.001) and 23.15 pmol/min.mL (95% CI: 10.70 to 35.59 pmol/min.mL; *p* value = 0.0004), respectively. No significant correlations were observed between BMI and postprandial changes in sEH in either group.

### 2.5. Associations of sEH with Anxiety and Depression

**Figure 6:**
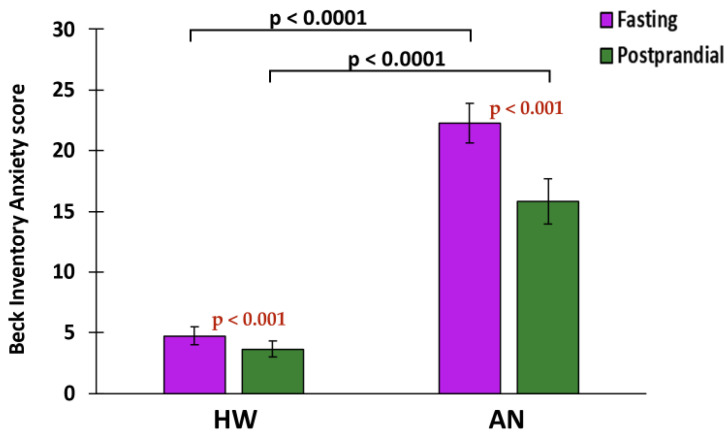
Bar plots for anxiety level at fasting (purple) and postprandial (green) timepoints in HW and AN.

HW displayed a lower fasting anxiety level compared to women with AN (4.74 ± 7.06 versus 22.27 ± 13.38, *p* value < 0.0001). At the two-hour postprandial timepoint, anxiety level remained significantly lower in HW (3.64 ± 6.50 versus 15.82 ± 11.50, *p* value < 0.0001, Table 1). After eating the study meal, both groups showed a significantly lower anxiety score. The postprandial anxiety score decrease was 1.20 ± 2.83 points for HW (*p* value < 0.0001) and 4.32 ± 7.23 points for women with AN (*p* value < 0.0001) (Figure 6).

**Figure 7:**
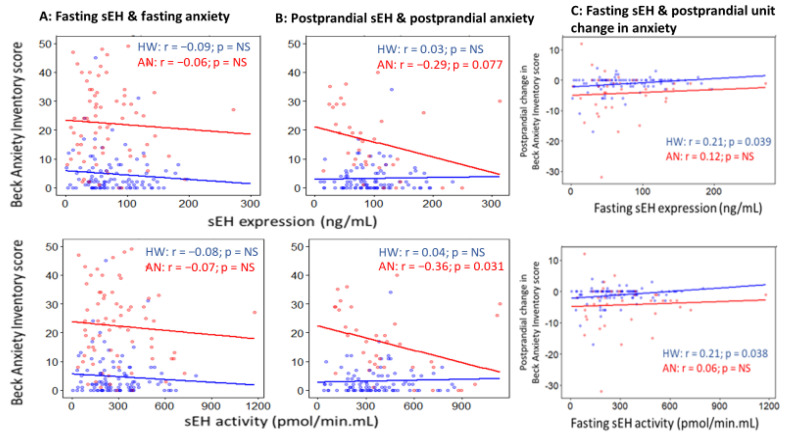
Scatterplots for Pearson’s correlations of sEH expression (top) and activity (bottom) with Beck Anxiety Score at (**A**) fasting and (**B**) postprandial timepoints in healthy women (blue) and women with anorexia nervosa (red). Scatterplots for Pearson’s correlations of sEH expression (top) and activity (bottom) with postprandial unit change in Beck Anxiety Score are presented in (**C**). Colored dots and solid lines represent individual data points and slopes of the examined relationships, respectively. r and p indicate Pearson’s correlation coefficient and *p* value, respectively. HW: healthy women; AN: anorexia nervosa; NS: non-significant, *p* value ≥ 0.10.

In HW, no significant associations were found for sEH with anxiety at both timepoints (Figure 7A,B) or depression. Fasting sEH expression and activity were, however, both positively correlated with postprandial unit change in anxiety (sEH expression: r = 0.21; *p* value = 0.039. sEH activity: r = 0.21; *p* value = 0.038) (Figure 7C). After adjustment for age and BMI, each unit increase in fasting sEH expression and activity was linked to a 0.014-point (95% CI: 0.0006 to 0.03 point; *p* value = 0.040) and 0.004-point (95% CI: 0.0004 to 0.008 point; *p* value = 0.030) lower decrease in HW’s postprandial anxiety.

In women with AN, neither sEH expression nor activity was significantly correlated with depression or fasting timepoint anxiety (Figure 7A). At the postprandial timepoint, sEH expression and activity showed an inverse correlation with anxiety (r = −0.29; *p* value = 0.077 and r = −0.36; *p* value = 0.031, respectively, Figure 7B). Fasting sEH expression and activity were, however, not associated with postprandial unit change in anxiety (Figure 7C).

### 2.6. Race and sEH

In HW, sEH expression and activity were not significantly different between White women (*n* = 48) and non-White women (*n* = 48) at either the fasting or postprandial timepoint, with or without adjustment for age, BMI, and assay batch. As Asian women comprised most of the non-White group, sEH expression and activity were compared between White women (*n* = 48) and Asian women (*n* = 39) in the HW group. Neither sEH expression nor sEH activity was significantly different between Asian women and White women at either timepoint, with or without covariate adjustment (Table S1).

## 3. Discussion

The catalytic role of sEH in the metabolism of oxylipins, bioactive lipid mediators involved in various biological functions and diseases [3,14,68,69,70], renders sEH a potential risk modifier of these diseases. However, the impacts that sEH has on risk factors of the diseases and disorders remain unclear. To address this knowledge gap, sEH protein expression and enzymatic activity were characterized using direct measurements, at both fasting and postprandial timepoints, through a food challenge study design. The use of a tightly controlled meal intake protocol enabled the accurate assessment of sEH’s quantitative changes two hours after eating the meal. The comparison of sEH in two groups of adult women enabled the assessment of sEH in healthy women and in a clinical control group of women with anorexia nervosa (AN). Unique characteristics in women with AN such as low BMI, aversion toward high fat foods, and high rates of comorbid depression or anxiety disorders [71,72,73,74,75,76,77,78] make this group an informative contrast group to healthy women.

The mechanisms by which mRNA transcripts are translated into protein molecules involve multiple regulatory factors and processes that affect mRNA and protein expression, and/or protein activity. For example, an enzyme’s stability, location, or specificity can be altered by its interaction with other proteins or with its own mRNA transcripts, phosphorylation, and/or the gut microbiota [16,46,79,80]. Despite these complex dynamics, a protein’s expression and activity are usually assumed to be correlated without further verification. For sEH, a concordance between sEH transcription and translation products was implied when in vitro *EPHX2* mRNA, sEH protein expression, and in vivo sEH activity were concordantly upregulated during adipogenesis [47]. However, in another study, sEH protein expression was found to be higher but the activity was lower when comparing mice of a depression model to wild-type mice [64]. The correlation between sEH protein expression and enzymatic activity in humans has not been published to date. We filled this gap by testing both the protein expression and enzymatic activity of sEH using direct measurement assays.

We found that sEH protein expression and sEH activity are significantly and highly correlated in both healthy women and women with AN (r = 0.91 and 0.92, *p* value < 0.001), despite the significant differences in BMI and other characteristics between groups (Table 1). Consumption of the high-fat study meal did not significantly alter the correlation between sEH expression and activity in either group (r = 0.89 and 0.90, *p* value < 0.001). For each unit increase in sEH expression, sEH activity increased by 3.4 pmol/min.mL in HW and 3.7 pmol/min.mL in women with AN at the fasting timepoint, and by 3.6 pmol/min.mL in both groups at the postprandial timepoint. We have confirmed that sEH protein abundance has a direct influence on its in vivo activity regardless of recent exposure to a high-fat meal.

While correlation between sEH expression and activity remains high at both fasting and postprandial timepoints, consumption of the high-fat study meal did increase sEH activity by 111% in HW and 56% in women with AN. Our data in HW are in full agreement with another study that found sEH mRNA expression upregulated by 1.23 times in obese individuals after eating a meal [17]. Many more studies using animal models have published data on the impact of a high-fat diet on sEH levels, but the results are mixed. Three rodent studies found elevated sEH protein expression and/or activity in mice fed a high-fat diet [18,19,20], whereas two studies found no significant difference for sEH gene expression resulting from two diets of differing fat-levels [9,81]. One study used three epoxide/diol ratios as sEH proxy markers and found only one linoleic acid-derived marker (9,10-EpOME/9,10-DiHOME) to suggest a decreased sEH activity in mice fed a high-fat diet [82]. In another study, sEH activity normalized by body weight was significantly lowered in the kidneys but was increased in the adipose tissue of mice fed a high-fat diet [9]. Discrepant findings on the effects of high-fat diet on sEH may be attributed to variation in the diet [19], disease/metabolic status [83], the host’s gut microbiota [16], tissue type [9], sEH detection method [9], and study design. The oxylipin ratios, commonly used as sEH activity in vivo markers, are vulnerable to rapid changes due to factors such as phospholipid remodeling, varying rates of oxylipin synthesis or degradation, and assay processing [65,66,67]. Our results, measured using direct sEH expression and activity assays, gave high confidence that the observed sEH increases after the consumption of a high-fat meal in adult women are valid.

Examining the relationships of sEH with age and BMI clarifies the effects that sEH has on these common “risk factors” of medical and psychiatric disorders. Aging is an inevitable process during which various biological functions are expected to decline. Knowledge of how sEH changes during the normal aging process can improve our understanding of both sEH mechanism and disease susceptibility. Our results in healthy women show that fasting sEH and postprandial sEH changes were not correlated with age. Our finding in HW is supported by data in three animal model papers which show that in wild-type mice, brain and cardiac sEH protein expression or activity did not change with age [50,51,52]. By contrast, both sEH expression and activity were inversely associated with age in women with AN. AN is a deadly eating disorder that commonly onsets in adolescence [84]. AN symptoms can improve when patients successfully respond to treatments and learn strategies to manage their lifetime triggers and symptoms as they grow older. The AN progression may contribute to the association between younger age and higher sEH levels. The correlation between age and sEH in AN may also result from hormonal abnormalities, such as estrogen deficiency and delayed puberty that were observed in AN [85,86,87], as estrogen has been shown to suppress vascular and cerebral sEH levels [88,89,90]. The differential findings in this clinical control group highlight the importance of considering the unique phenotypes in the disordered population.

BMI is a well-studied risk factor for cardiometabolic disorders [28,31,32,39,40]. Several studies found that sEH expression and activity were increased in obese humans, in mice, and during adipogenesis [9,42,43,44,45,46,47,48,49]. Our earlier work has shown that a *EPHX2* polymorphism, rs2291635, moderated the association between BMI increase and cholesterol levels [91]. In this study, despite significant BMI differences between HW and AN groups, BMI was positively correlated with sEH expression and activity in both groups, consistent with previous findings that higher BMI is linked to a higher level of sEH [9,42,43,44,45,46,47,48,49].

Anxiety and depression are prevalent disorders that have been exacerbated by the COVID-19 pandemic, with 30.7–42.4% of adults in the United States reporting anxiety or depressive symptoms [92,93]. This is a striking increase from the pre-pandemic prevalence rate of 11% [92,93]. At the beginning of the study visit, anxiety level was significantly higher in women with AN, reflecting the comorbid anxiety as well as anticipatory anxiety associated with eating in AN [94]. Anxiety dropped in both HW and AN groups two hours after eating the study meal, possibly due to the “relief” the study participants felt after having finished the meal. The degree of postprandial anxiety decrease was greater in women with AN than in HW in part due to their higher baseline anxiety. sEH was not significantly associated with anxiety in either group at the fasting timepoint, yet sEH was associated with postprandial anxiety differently in the HW and AN groups. These differential associations are likely due to significant between-group differences in anxiety severity, variability in the postprandial increase in sEH, and sample size difference at the postprandial timepoint. Two studies using animal models have suggested that sEH may play a role in anxiety, as sEH knock-out induced anxiety-like behaviors [62,63]. Further work is needed to understand the link between sEH and anxiety, especially in human patients.

We did not uncover any significant associations between sEH and depression in either group. Reports in animal model studies, however, have shown high sEH expression under chronic stress and depressive conditions [58,59,60], and that sEH knock-out appears to exert anti-depressive effects [59,60,61,64]. In diabetic patients, sEH proxy markers did not differ significantly between depressed and non-depressed patients, with the exception of one proxy marker (12,13-DiHOME/12,13-EpOME) [70]. Just like anxiety, depression is a complex phenotype that ranges from a mild, transient state to a treatment-resistant/refractory clinical disorder [95]. Heterogeneity in the manifestation of depression and anxiety means that a significantly larger population-based sample size is required to detect a biomarker association.

Previous studies reported that several *EPHX2* and Cytochrome P450 (CYP) genetic polymorphisms showed different allele frequencies in different racial groups [96,97,98], suggesting that race may be a determinant of sEH level. We found no significant differences in sEH levels between White women and non-White women, as well as between White women and Asian women (largest non-White group). However, our sample size in each race group is too small to rule out race effect on sEH, quantitatively or qualitatively.

The strengths of our study include being the first to confirm by direct assays the significant correlation between sEH protein expression and enzymatic activity, and to characterize sEH quantitatively across different timepoints in healthy and clinical groups. The direct measurement of sEH activity (instead of using oxylipin ratios as proxy markers) allowed us to isolate sEH’s action from biological processes that are oxylipin specific. Examining both sEH activity and expression in healthy and clinical control groups enables a comprehensive assessment of sEH in different states of human health. Furthermore, we demonstrated the effect that a high-fat meal has on sEH using a well-controlled food challenge protocol to ensure validity and reproducibility. This study was limited by the smaller AN sample size at the postprandial timepoint due to clinical participants’ refusal to eat the study meal. Another weakness is the study’s focus on sEH in women only. However, this study design allowed us to remove hormonal and sex-specific confounders. Given the differential distribution [11,12,13,99] and hormonal regulation of sEH in organs and tissue types [89,90,100,101], our blood-derived sEH data may not accurately reflect the sEH level of a specific organ. Thus, future sEH research may benefit from disease-specific tissue samples to better understand disease pathogenesis.

In summary, we observed a strong positive correlation between sEH protein expression and enzymatic activity in both healthy women and women with AN. We showed that fasting sEH was positively associated with BMI in both groups, and that sEH was inversely associated with age in women with AN only. While sEH had no significant association with depression or anxiety at the fasting timepoint in both groups, sEH’s association with anxiety in women with AN after eating highlights the need for phenotype-dependent considerations in biomarker research. Together, our findings revealed sEH’s potential to modify common risk factors of human diseases/disorders. Our data provide new knowledge of sEH in adult women of good health through a comparison to women with an eating disorder. To conclude, the direct assessment of sEH, as presented in this paper, should be encouraged in future studies to ensure accuracy and validity.

## 4. Materials and Methods

### 4.1. Participants and Study Design

Healthy women (HW) and women with anorexia nervosa (AN) were recruited from the University of California San Diego, the San Diego region of California, USA, University of Toronto, and the city of Toronto, Canada. Exclusion criteria for all study participants included Axis I psychiatric illness, organic brain syndrome, schizophrenia or schizoaffective disorder, untreated thyroid disease, renal disease, hepatic disease, pregnancy or breast-feeding, and the regular use of fish oil supplements.

A total of 96 healthy women (18–53 years old; mean age: 29 ± 8) and 70 women with AN (18–68 years old; mean age: 32 ± 12) were enrolled in the study. On the day of the study visit, each study participant had her weight and height measured by a clinical nurse. After at least 10 h of fasting, all participants completed the Beck Depression Inventory and Beck Anxiety Inventory questionnaires and donated blood samples that directly underwent peripheral blood mononuclear cell (PBMC) extraction for sEH assays. The participants then ate the study meal, which was an egg-and-sausage muffin containing 436 calories, 27 g of fat, 19 g of protein, and 28.5 g of carbohydrates. Ninety-two healthy women and 38 women with AN completed the study meal, filled out the postprandial Beck Anxiety Inventory questionnaire and donated postprandial blood samples two hours after consumption of the study meal. Reasons for the participants’ unwillingness to consume the meal or donate postprandial blood samples included fear of the study meal and difficulty in the blood draws. This study has been approved by both the University of California-San Diego (UCSD) Human Protection Board and the University of Toronto Research Ethics Board.

### 4.2. sEH Protein Level Quantification

The sEH level in PBMCs was measured using an ultrasensitive PolyHRP based immunoassay, as described elsewhere [102]. Briefly, the high-binding microplate (Nunc Cat.No. 442404) was coated with anti-human sEH rabbit serum (1:2000 dilution) in 0.05 M pH 9.6 carbonate-bicarbonate buffer (100 μL/well) overnight at 4 °C. After washing, the plate was blocked with 3% (*w*/*v*) skim milk (300 μL/well) in PBS for one hour and washed before sample application. Serial concentrations of human sEH standards or samples with different dilutions in PBS containing 0.1 mg/mL bovine serum albumin (BSA) were then added to the wells (100 μL/well). To minimize the variation of incubation, human sEH standards and samples were first loaded into a 2-mL 96 deep well microplate (Costar 3960), and then transferred to the microplate for immunoassay within 1 min using a multi-channel pipette. Immediately, biotinylated nanobodies selected for reaction with the human sEH (1 μg/mL, 100 μL/well) in PBS were added to each well. The immunoreaction was allowed to proceed for 1 h. After washing, SA-PolyHRP in PBS (25 ng/mL, 100 μL/well) was added and the reaction continued for another 30 min. After the final washing, 3,3′,5,5′-tetramethylbenzidine (TMB) substrate (100 μL/well) was added and the plate was incubated for 10–15 min. After stopping the color development with 2 M of sulfuric acid (100 μL/well), the optical density was recorded at 450 nm within 10 min. All incubations unless otherwise specified were performed at room temperature with shaking (600 rpm); each washing step involved three washings with PBS containing 0.05% Tween-20 (PBST, 300 μL/well). Since the reaction involved two separate sEH-targeting antibodies, one of which was a monoclonal nanobody, the assay was exceptionally selective and sensitive.

### 4.3. sEH Cellular Activity

Peripheral blood mononuclear cells (PBMCs) were immediately isolated by density gradient centrifugation with Ficoll-Paque (GE Healthcare Bio-Sciences AB, Uppsala, Sweden). The cell suspensions were flash-frozen and kept at −80 °C until analysis. After thawing on ice, the cells were broken with a 10-second ultrasonic pulse. Protein concentration was quantified using the Pierce BCA assay (Pierce, Rockford, IL, USA), using Fraction V bovine serum albumin (BSA) as the calibrating standard. Separately, the homogenized mixture was diluted with chilled sodium phosphate buffer (20 mM pH 7.4) containing 5 mM of EDTA, 0. 1 mM of DTT, 1 mM of PMSF, 0.1 mg/mL of BSA and 0.01% Tween 20 to measure the residual sEH activity using [^3^H]-trans-diphenyl-propene oxide (t-DPPO) as substrate [103]. Briefly, 1 µL of a 5 mM solution of t-DPPO in DMSO was added to 100 µL of diluted homogenate ([S]_final_ = 50 µM). The mixture was incubated at 37 °C for 90 min, and the reaction was quenched by the addition of 60 µL of methanol and 200 µL of isooctane, which extracts the remaining epoxide from the aqueous phase. Extractions of the stopped reaction with 200 µL of 1-hexanol were performed in parallel to assess the possible presence of glutathione transferase activity, which could also transform the substrate [103]. The activity was followed by measuring the quantity of radioactive diol formed in the aqueous phase using a scintillation counter (TriCarb 2810 TR, Perkin Elmer, Shelton, CT, USA). Assays were performed in triplicate.

### 4.4. Statistical Analysis

Correlations between sEH expression and activity were analyzed in HW and women with AN using Pearson’s correlations and multiple linear regression models that were adjusted for age and BMI. sEH protein expression and enzymatic activity, and their postprandial changes, were compared between the two groups using two-sample *t*-tests and analysis of covariance (ANCOVA) models that were adjusted for age, BMI, and sEH assay batch. Postprandial changes in sEH were assessed using one-sample *t*-tests within each group separately. sEH associations with age, BMI, and clinical phenotypes were examined within each group using Pearson’s correlations as well as age-and-BMI-adjusted linear regression models. The cell-count-normalized data of sEH expression and activity were used to verify the validity of the results of non-normalized (absolute) sEH levels. In HW only, sEH expression and activity were compared between White women and non-White women, as well as between White women and Asian women, at the fasting and postprandial timepoints using two-sample *t*-tests and ANCOVA models that were adjusted for age, BMI, and sEH assay batch. All statistical analysis was performed using R version 4.2.1 (R Foundation for Statistical Computing, Vienna, Austria).

## Figures and Tables

**Table 1:** Study participant characteristics.

Characteristic	Healthy Women *n* = 96	Anorexia Nervosa *n* = 70
Age (years)	29 ± 8 (95% CI: 27 to 30)	32 ± 12 (95% CI: 29 to 34)
Race: n (%)	White: 48 (50%) Asian: 39 (41%) Black/African American: 2 (2%) More than one race: 7 (7%)	White: 62 (89%) Asian: 7 (10%) Black/African American: 0 (0%) More than one race: 1 (1%)
Body mass index (kg/m^2^)	22.98 ± 3.16 * (95% CI: 22.34 to 23.62)	18.66 ± 3.44 (95% CI: 17.84 to 19.48)
Beck Depression Inventory score (fasting)	4.05 ± 6.22 * (95% CI: 2.79 to 5.31)	22.900 ± 15.75 (95% CI: 19.14 to 26.66)
Beck Anxiety Inventory Score (fasting)	4.74 ± 7.06 * (95% CI: 3.31 to 6.17)	22.27 ± 13.38 (95% CI: 19.08 to 25.46)
Postprandial Beck Anxiety Inventory Score	3.64 ± 6.50 * (95% CI: 2.31 to 4.97)	15.82 ± 11.50 (95% CI: 12.03 to 19.60)
Postprandial unit change in Beck Anxiety Inventory Score *	−1.20 ± 2.83 (95% CI: −1.78 to −0.62)	−4.32 ± 7.23 (95% CI: −6.69 to −1.94)

Note: Data are reported in mean ± standard deviation (95% Confidence interval [CI]). * represents *p* value < 0.05 comparing healthy women and anorexia nervosa groups.

## Supplementary Materials

The following supporting information can be downloaded at: https://www.mdpi.com/article/10.3390/ijms231911798/s1.
