# Supplementary material for: Soluble Epoxide Hydrolase Is Associated with Postprandial Anxiety Decrease in Healthy Adult Women
Source: Int J Mol Sci. 2022 Oct 5;23(19):11798. doi: 10.3390/ijms231911798 (PMC9569757; doi:10.3390/ijms231911798)
Supplement: Supplementary file 1 [file ijms-23-11798-s001.zip › ijms-1910468-supplementary.pdf]

Table S1. sEH expression (ng/mL) and sEH activity (pmol/min.mL) across different racial groups in healthy women at fasting and postprandial timepoints

Between-group comparison	sEH Expression (ng/mL)		sEH Activity (pmol/min.mL)	
Race	Mean ± SD (ng/mL)	Unadjusted p-value; adjusted p-value	Mean ± SD (pmol/min.mL)	Unadjusted p-value; adjusted p-value
White (n=48)	F: 76.00 ± 40.10<br>P: 101.93 ± 49.69	—	F: 288.13 ± 154.83<br>P: 384.87 ± 193.19	—
Asian (n=39)	F: 77.42 ± 43.41<br>P: 86.34 ± 39.00	F: 0.874; 0.962<br>P: 0.121; 0.181	F: 278.20 ± 153.37<br>P: 323.55 ± 183.23	F: 0.766; 0.672<br>P: 0.143; 0.135
Black or African American (n=2)	F: 117.56 ± 84.16<br>P: 123.94 ± 66.85	—	F: 506.33 ± 414.47<br>P: 511.51 ± 295.70	—
More than one race (n=7)	F: 52.04 ± 60.34<br>P: 93.38 ± 50.66	—	F: 194.26 ± 179.85<br>P: 341.59 ± 177.73	—

Note: Unadjusted p-values are obtained from two-sample t-tests comparing Asians against Whites. Adjusted p-values are from analysis of covariance (ANCOVA) models adjusted for age, BMI, and sEH assay batch. F: fasting; P: postprandial; SD: standard deviation.

Table S2. List of abbreviations

**Abbreviation**	**Definition**
AN	Anorexia nervosa
ANCOVA	Analysis of covariance
BMI	Body mass index
CI	Confidence interval
CYP	Cytochrome P450
HW	Healthy women
NS	Non-significant
PBMC	Peripheral blood mononuclear cells
sEH	Soluble epoxide hydrolase
